# Novel *ATXN1/ATXN1L::NUTM2A* fusions identified in aggressive infant sarcomas with gene expression and methylation patterns similar to *CIC-*rearranged sarcoma

**DOI:** 10.1186/s40478-022-01401-z

**Published:** 2022-07-14

**Authors:** Feng Xu, Angela N. Viaene, Jenny Ruiz, Jeffrey Schubert, Jinhua Wu, Jiani Chen, Kajia Cao, Weixuan Fu, Rochelle Bagatell, Zhiqian Fan, Ariel Long, Luca Pagliaroli, Yiming Zhong, Minjie Luo, Portia A. Kreiger, Lea F. Surrey, Gerald B. Wertheim, Kristina A. Cole, Marilyn M. Li, Mariarita Santi, Phillip B. Storm

**Affiliations:** 1 Department of Pathology and Laboratory Medicine, Children’s Hospital of Philadelphia, Philadelphia, PA USA; 2 Department of Pediatrics, Children’s Hospital of Philadelphia, Philadelphia, PA USA; 3 Perelman School of Medicine, University of Pennsylvania, Philadelphia, PA USA

**Keywords:** *CIC-*rearranged sarcoma, *ATXN1/ATXN1L*-associated fusions, Whole transcriptome sequencing

## Abstract

*CIC*-rearranged sarcomas are newly defined undifferentiated soft tissue tumors with *CIC-*associated fusions, and dismal prognosis. *CIC* fusions activate PEA3 family genes, *ETV1/4/5,* leading to tumorigenesis and progression. We report two high-grade CNS sarcomas of unclear histological diagnosis and one disseminated tumor of unknown origin with novel fusions and similar gene-expression/methylation patterns without *CIC* rearrangement. All three patients were infants with aggressive diseases, and two experienced rapid disease deterioration and death. Whole-transcriptome sequencing identified an *ATXN1-NUTM2A* fusion in the two CNS tumors and an *ATXN1L-NUTM2A* fusion in case 3. *ETV1/4/5* and *WT1* overexpression were observed in all three cases. Methylation analyses predicted *CIC*-rearranged sarcoma for all cases. Retrospective IHC staining on case 2 demonstrated ETV4 and WT1 overexpression. ATXN1 and ATXN1L interact with CIC forming a transcription repressor complex. We propose that *ATXN1/ATXN1L*-associated fusions disrupt their interaction with CIC and decrease the transcription repressor complex, leading to downstream PEA3 family gene overexpression. These three cases with novel *ATXN1/ATXN1L*-associated fusions and features of *CIC*-rearranged sarcomas may further expand the scope of “*CIC*-rearranged” sarcomas to include non-*CIC* rearrangements. Additional cases are needed to demonstrate if *ATXN1/ATXN1L-NUTM2A* fusions are associated with younger age and more aggressive diseases.

## Introduction

*CIC*-rearranged sarcomas are a group of newly defined high-grade undifferentiated small round cell soft tissue tumors with *CIC-*associated fusions, most often *CIC*::*DUX4* [1]*.* These tumors were previously classified as Ewing sarcoma family of tumors (EFTs) but have a markedly worse prognosis compared to that of EFTs without a *CIC* rearrangement [2, 3]. *CIC* fusions activate PEA3 family genes, *ETV1/4/5,* leading to tumorigenesis and progression. Siegfried et al. first reported an *ATXN1::NUTM1* gene fusion in a primitive brain tumor in a 21-year-old woman in 2019. The fusion gene transcript encompassed almost all of the *ATXN1* coding sequence and the exon 6, 7 and 8 regions of *NUTM1*. Methylation profiling predicted the tumor to be a CNS Ewing sarcoma family tumor with *CIC* alteration with low confidence [4]. Pratt et al. recently reported a CNS sarcoma characterized by an *ATXN1::DUX4* fusion with PEA3 family gene overexpression in a 3-year-old boy. The methylation array also placed this tumor within the *CIC*-rearranged sarcoma group [5]. The authors proposed to expand the spectrum of '*CIC*-rearranged sarcoma' of the CNS to include non-*CIC* alterations [5].

We report three pediatric sarcomas, including two high-grade central nervous system (CNS) sarcomas and one disseminated tumor of unknown origin, with novel fusions involving *ATXN1/ATXN1L* and gene-expression/methylation patterns similar to that of *CIC*-rearranged sarcomas in the absence of *CIC-*associated fusions.

## Case presentation

**Fig. 1:**
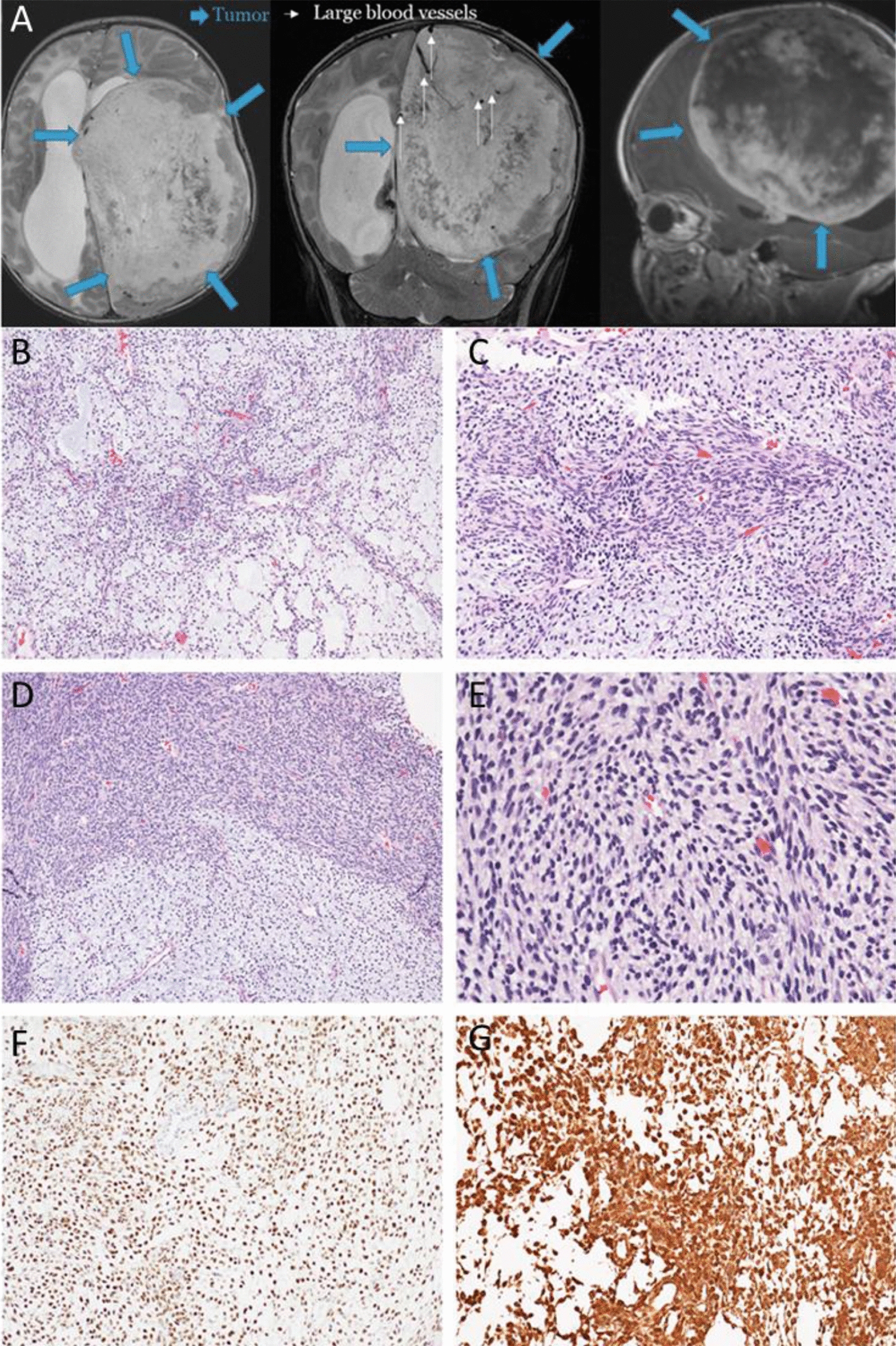
MRI and histologic findings of case 1 with *ATXN1::NUTM2A* fusion. **A** MRI: T2WI—axial and coronal; T1WI axial, post gadolinium images showed a large mixed solid and cystic mass expanding the left cerebral hemisphere, rightward midline shift, and marked flattening and displacement of the brainstem and cerebellum [Blue arrows outline the tumor mass; white arrows indicate large blood vessels]. **B** The tumor demonstrates prominent myxoid stromal changes with reticular arrangement of cells. **C**, **D** The tumor showed interfaced nodules and diffuse sheets of undifferentiated round to ovoid cells. **E** The tumor cells showed a relatively uniform cytomorphology at a higher magnification. **F**, **G** The tumor cells are positive for ETV4 (**F**) and vimentin (**G**) by immunohistochemistry. (**B**, **C**, **D** and **E**: hematoxylin and eosin [**H**&**E**], 100x, 200 × and 400 × final magnification; F and G: ETV4 and vimentin, 200 × final magnification)

Case 1 was an eight-week-old male infant who presented with irritability and increasing head circumference. Brain MRI showed a large mixed solid and cystic mass markedly expanding the left cerebral hemisphere (Fig. 1A). The associated mass effect resulted in rightward midline shift, uncal and subfalcine herniation, and marked flattening and displacement of the brainstem and cerebellum. The patient underwent craniotomy for subtotal tumor resection. Histologic examination demonstrated a tumor with alternating regions of solid and looser, microcystic growth (Fig. 1B–D). The looser regions contained a myxoid-rich background and cells with round to ovoid nuclei and fine chromatin. The solid component demonstrated multiple growth patterns including organoid, fascicular, whorled appearances. Within the regions of solid growth, the tumor cells were ovoid to spindled with fine chromatin, a small amount of pale eosinophilic cytoplasm, mild nuclear atypia, and inconspicuous nucleoli (Fig. 1E). There was no microvascular proliferation and no necrosis. Ten mitoses were counted in ten high power fields. The proliferative index by Ki-67 immunostain was approximately 30–40%. The tumor cells were positive for vimentin (Fig. 1G). INI-1 and ATRX were retained. BRG-1 was retained in the vessels but was lost in the majority of tumor nuclei. BCL6 demonstrated focal reactivity. SATB2, GFAP, Olig2, synaptophysin, neurofilament, H3K27M, and IDH1-R132H were negative. An immunostain for ETV4 showed diffuse nuclear positivity (Fig. 1F). Despite aggressive medical interventions, the patient expired on day of life 57.

Case 2 was a sixteen-month-old male who presented with seizures, lethargy, and vomiting. A brain MRI showed a large left frontal cystic and solid hemorrhagic mass with dural attachment and midline shift. The patient underwent craniotomy, and a gross total resection was achieved. Microscopic examination showed a tumor with alternating regions of high and moderate cellularity with focal myxoid background. The histologic pattern was variable with fascicular, nodular, and cord-like areas. The majority of the tumor cells were small with round to oval nuclei, fine chromatin, inconspicuous nucleoli, and a small amount of pale eosinophilic cytoplasm. Rare nests of cells with large nuclei with conspicuous nucleoli were present. Small foci of non-palisading necrosis were seen, and up to nine mitoses were counted in ten high power fields. Tumor cells were positive for vimentin, and INI-1 was retained. Neuronal and glial markers were positive only in a small subset of cells. Cytogenetic and chromosomal SNP Array analyses on the tumor showed a normal male complement. The patient was treated with multi-agent chemotherapy including high dose chemotherapy with stem cell rescue, and off therapy imaging showed no evidence of disease. Approximately two months off therapy, and nine months after initial diagnosis, the patient experienced progressive emesis and lethargy. MRI imaging revealed a new large complex mass measuring 6.1 × 6.4 × 3.4 cm in the left inferior frontal resection site, consistent with tumor recurrence. His general condition deteriorated quickly, and he was admitted to Hospice Services and is under palliative care.

Case 3 was a 30-week-gestation male neonate with a disseminated tumor of unknown origin. At birth, he had numerous blue-purple skin nodules throughout his body, generalized edema, and severe anemia concerning for hydrops fetalis. An abdominal ultrasound showed a 2.4 cm left upper quadrant mass encasing an adjacent bowel loop and abutting, but not invading the adrenal gland. MRI detailed multiple soft tissue masses throughout the retroperitoneum and pelvis. Microscopic examination from a skin biopsy demonstrated a cellular infiltrate composed of primitive small round cells with a high nuclear-cytoplasmic ratio. An extensive panel of immunohistochemistry stains was negative except for diffusely positive CD99, patchy positive vimentin, and focally positive CD117, GATA1 and TCL1. INI1 was retained. Rare morphologically atypical cells were seen within the peripheral blood; however, flow cytometry was negative for a hematopoietic neoplasm. Cytogenetic analysis of the peripheral blood showed a balanced t(10;16) (q24;q24) in 4 of 43 metaphases, ultimately considered to be circulating tumor cells. FISH analysis on the peripheral blood was negative for *KMT2A, PML/RARA,* or *CBFB* gene rearrangements. Additional FISH on the skin lesion was negative for rearrangements of *KMT2A, GLIS2*, and *CREBBP*. The neonate developed multisystem organ failure despite aggressive medical interventions and expired on day 14 of life. Post-mortem examination showed that the tumor encased and infiltrated nearly every thoracic and abdominal organ. Tumor encased the entire spinal cord with subarachnoid spread within the posterior fossa causing obstructive hydrocephalus and marked thinning of the cortical mantle. The microscopic morphology was similar to the pre-mortem skin biopsy: round cells with a high nuclear-cytoplasmic ratio, vesicular nuclear chromatin with small nucleoli, and scant clear to eosinophilic cytoplasm. Up to 37 mitoses were counted in 10 high power fields. A myxoid background and different growth patterns were not appreciated despite extensive sampling of the tumor. Stains for AE1/3, GFAP, and synaptophysin were performed on the tumor involving the posterior fossa and were negative; vimentin was patchy positive.

**Fig. 2:**
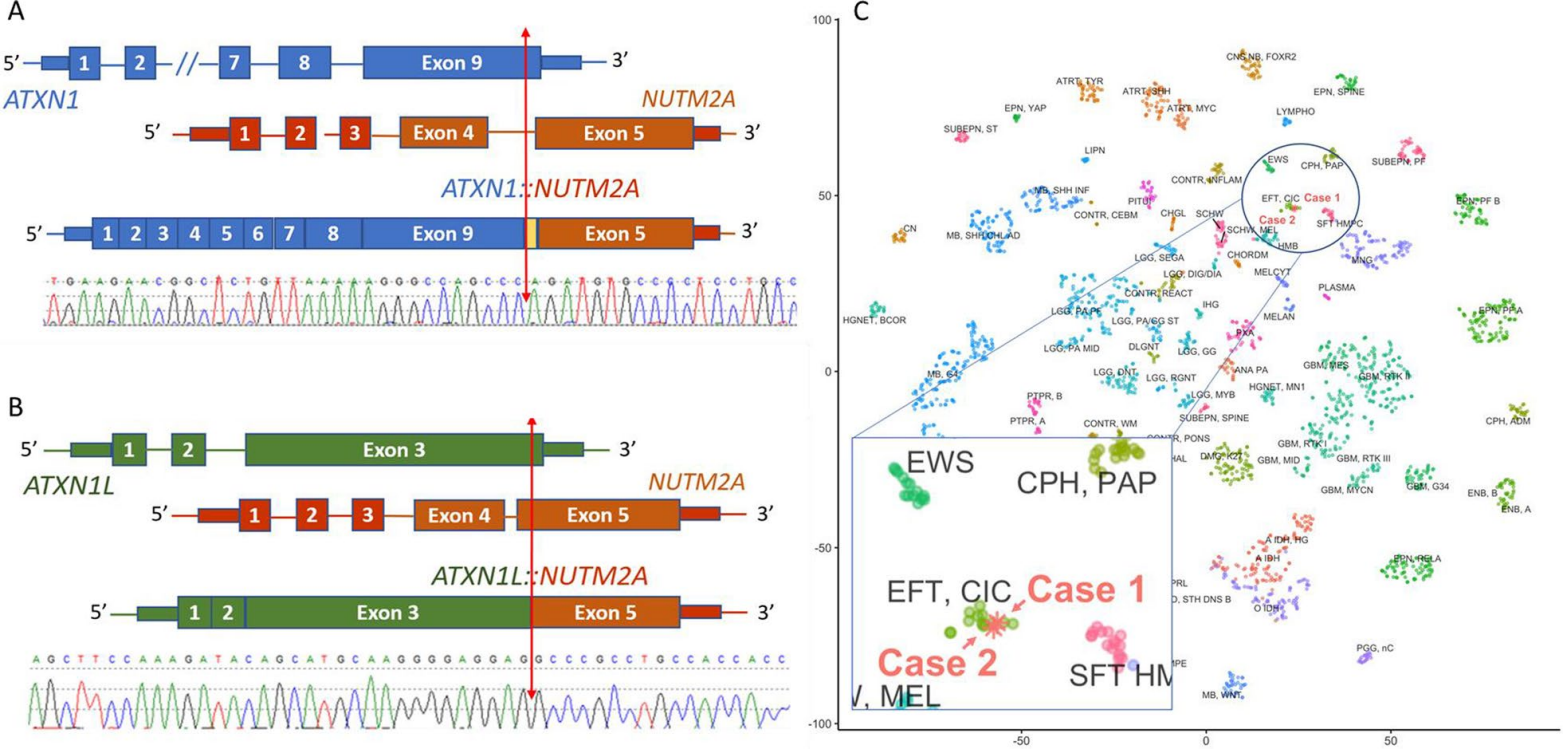
**A** Ideogram and Sanger confirmation of case 1: *ATXN1::NUTM2A* (blue: *ATXN1*, orange: *NUTM2A*, yellow: intron 4 linker sequence); **B** Ideogram and Sanger confirmation of *ATXN1L::NUTM2A* (green: *ATNX1L*, orange: *NUTM2A*); **C** t-SNE plot showing the methylation results of the two CNS sarcomas grouped together with *CIC*-rearranged sarcoma group

A comprehensive next generation sequencing panel analysis that interrogates 238 cancer genes for mutations and copy number alterations, and 117 cancer genes for fusions were performed on the three tumors but failed to identify genomic evidence for tumor diagnosis [6, 7]. Whole-transcriptome sequencing (RNA-seq) identified a novel fusion *ATXN1::NUTM2A* in both cases 1 and 2 with slightly different breakpoints in both genes. Though the breakpoints in *ATXN1* are different, both are in the last exon of *ATXN1* and distal to the AXH domain. The fusion in case 1 included a 160 bp intronic sequence from intron 4 of *NUTM2A* as a linker leading to an inframe fusion (Fig. 2A). In case 3, RNA-seq identified a novel fusion *ATXN1L::NUTM2A* with the breakpoint in *ATXN1L* also in the last exon distal to the AXH domain (Fig. 2B). Additionally, RNA-seq demonstrated the overexpression of *ETV1/4/5* in all three cases, similar to the gene expression pattern reported in *CIC*-rearranged sarcomas [5]. Since *CIC*-rearranged sarcomas were all positive for ETV4 by IHC staining [8], we performed retrospective IHC on tissue from case 1. This demonstrated ETV4 protein overexpression (Fig. 1F). Methylation profiling using Illumina Human MethylationEPIC BeadChip (Illumina, Inc. San Diego, CA) classified cases 1 and 2 as *CIC*-rearranged CNS sarcoma with high confidence scores of 0.9997 and 0.99998, respectively (DKFZ CNS Tumor Classifier v12.5) (Fig. 2C) [9]. Methylation analysis for case 3 also predicted a *CIC*-rearranged sarcoma with a much lower confidence score (0.18945, DKFZ CNS Tumor Classifier v12.5), most likely due to the DNA used for the methylation study being extracted from a skin lesion.

The new 2021 WHO Classification of Tumors of the CNS states that all *CIC*-rearranged sarcomas, irrespective of location, uniformly contain an oncogenic gene fusion of a *CIC* transcriptional repressor with various partners [2, 4, 10–14];. We report three patients with aggressive sarcomas with gene expression and methylation patterns similar to that of *CIC*-rearranged sarcomas without a *CIC*-related fusion. Instead, a novel fusion involving *ATXN1* or *ATXN1L* was identified in each case. Pratt et al. recently reported a CNS sarcoma with *ATXN1::DUX4* fusion with PEA3 family gene overexpression in a 3-year-old boy. The methylation array also placed this tumor within the *CIC-*rearranged sarcoma group [5]. The ATXN1/ATXN1L protein forms a transcriptional repressor complex with capicua (CIC), and CIC anchors the complex to DNA, repressing its target genes [15]. The ATXN1/ATXN1L-CIC complex is essential to normal brain development. An in vivo study showed that knocking-out *atxn1* in mice destabilized *cic,* leading to de-repression of its target genes including PEA3 gene family members (*ETV1/4/5*) [5, 10, 16, 17]. We hypothesize that *ATXN1/ATXN1L*-associated fusions alter the protein structure of ATXN1/ATXN1L and destabilize the ATXN1/ATXN1L-CIC transcriptional repressor complex*,* leading to downstream gene overexpression and tumorigenesis. The close functional bond of proteins ATXN1, ATXN1L and CIC, and the additional three cases with *ATXN1/ATXN1L*-associated fusions reported here support expanding the *CIC-*rearranged sarcoma entity to include *ATXN1/ATXN1L*-rearranged sarcomas ("*CIC*-altered sarcomas” as suggested by Pratt et al. [5]). More cases are needed to further define the similarities and differences of these non-*CIC* rearranged sarcomas compared to *CIC*-rearranged sarcomas and the potential clinical impact of different fusion partners.

In summary, we report three aggressive undifferentiated sarcomas in infants or very young children with novel *ATXN1/ATXN1L*-associated fusions and gene-expression and methylation patterns similar to that of *CIC*-rearranged sarcomas. Our findings support expanding the scope of “*CIC*-rearranged” sarcoma to include non-*CIC* alterations. Additional cases are needed to demonstrate if *ATXN1/ATXN1L::NUTM2A* fusions are associated with patients of younger age and more aggressive disease.
